# MicroRNA-30a-5p promotes differentiation in neonatal mouse spermatogonial stem cells (SSCs)

**DOI:** 10.1186/s12958-021-00758-5

**Published:** 2021-06-09

**Authors:** Maryam Khanehzad, Seyed Mehdi Nourashrafeddin, Farid Abolhassani, Shokoofeh Kazemzadeh, Soheila Madadi, Elham Shiri, Parastoo Khanlari, Zahra Khosravizadeh, Azim Hedayatpour

**Affiliations:** 1 Department of Anatomy, School of Medicine, Tehran University of Medical Science, Tehran, Iran; 2 Department of Obstetrics, Gynecology and Reproductive Sciences, School of Medicine, University of Pittsburgh, Pittsburgh, USA; 3 School of Advanced Technologies in Medicine, Tehran University of Medical Sciences, Tehran, Iran; 4 Department of Anatomy, School of Medicine, Arak University of Medical Science, Arak, Iran; 5 Department of Anatomical Sciences, School of Medicine, Hamadan University of Medical Sciences, Hamadan, Iran

**Keywords:** Spermatogonial stem cell (SSC), miRNA-30a-5p, Differentiation

## Abstract

**Background:**

The importance of spermatogonial stem cells (SSCs) in spermatogenesis is crucial and intrinsic factors and extrinsic signals mediate fate decisions of SSCs. Among endogenous regulators, microRNAs (miRNAs) play critical role in spermatogenesis. However, the mechanisms which individual miRNAs regulate self- renewal and differentiation of SSCs are unknown. The aim of this study was to investigate effects of miRNA-30a-5p inhibitor on fate determinations of SSCs.

**Methods:**

SSCs were isolated from testes of neonate mice (3–6 days old) and their purities were performed by flow cytometry with ID4 and Thy1 markers. Cultured cells were transfected with miRNA- 30a-5p inhibitor. Evaluation of the proliferation (GFRA1, PLZF and ID4) and differentiation (C-Kit & STRA8) markers of SSCs were accomplished by immunocytochemistry and western blot 48 h after transfection.

**Results:**

Based on the results of flow cytometry with ID4 and Thy1 markers, percentage of purity of SSCs was about 84.3 and 97.4 % respectively. It was found that expression of differentiation markers after transfection was significantly higher in miRNA-30a- 5p inhibitor group compared to other groups. The results of proliferation markers evaluation also showed decrease of GFRA1, PLZF and ID4 protein in SSCs transfected with miRNA-30a-5p inhibitor compared to the other groups.

**Conclusions:**

It can be concluded that inhibition of miRNA-30a-5p by overexpression of differentiation markers promotes differentiation of Spermatogonial Stem Cells.

**Supplementary Information:**

The online version contains supplementary material available at 10.1186/s12958-021-00758-5.

## Background

Spermatogenesis is a complex developmental process in the mammalian reproductive system that results in generation of highly specialized sperm from spermatogonial stem cells (SSCs) [1, 2]. SSCs as the foundation cells are vital for spermatogenesis, but only form about 0.02–0.03 % of the total cell population in the adult mouse testis [3, 4]. SSCs have some outstanding characteristics compared to other human stem cells. First, they are the only stem cells with the capacity for transferring genetic information to next generations. Therefore, they are a promising resource for genetic modification experiments. Second, self-renewal throughout life and the ability of SSCs to differentiate can be utilized to clarify the signaling pathways leading to spermatogenesis. Third, SSCs can differentiate into all cell lineages based on their pluripotency capability [5, 6]. Therefore, SSCs can be used for cell therapy in reproductive medicine to treat infertility and for regenerative therapy to treat diseases without immune rejection [7]. The commitment of undifferentiated spermatogomia to differentiated spermatogonia and normal spermatogenesis requires the action of intrinsic agents and extrinsic signaling pathways [8, 9]. Recent studies have shown that microRNAs (miRNAs) are a class of endogenous factors involved in different cellular processes, including self-renewal, proliferation, differentiation, and apoptosis [10]. miRNAs are small single-stranded RNA molecules (18–25 nucleotides) that act as vital factors for post-transcriptional gene silencing. They bind to three untranslated regions of target mRNAs and result in either endonucleolytic cleavage of the target mRNA or translation inhibition [8, 11]. Several studies have found that can regulate the balance between proliferation and differentiation of SSCs and considered miRNAs as an important regulatory element for spermatogenesis [12, 13]. For example, the high expression of miRNA-100 in SSCs promotes their proliferation by STAT3 [13]. Moreover, it has been reported that the self-renewal of SSCs is regulated by MiRNA − 10b and MiRNA − 322 [4, 14]. Inhibition of cell cycle regulators and RNA binding proteins by MiRNA − 202 leads to the maintenance of spermatogonial stem cells [15]. MiRNA-34c enhances the differentiation of mouse spermatogonial stem cells by targeting Nanos2 [16]. MiRNA − 17–92 (Mirc1) and miRNA − 106b-25 (Mirc3) play key roles in the promotion of spermatogonial differentiation in mice [9]. Inhibition of the factors needed for maintaining the undifferentiated state results in the differentiation of undifferentiated spermatogonia. Differentiation of spermatogonia via RA leads to the suppression of LIN28; as a result, the Mirlet7 family is induced and further downregulates the genes associated with self-renewal of spermatogonia [17]. Nevertheless, the function and molecular mechanisms of individual miRNAs in regulating the SSCs fate determination are not clear warranting further research.

The miRNA-30 family as an important member of miRNA family contains five members and 6 mature miRNA molecules (namely, miR-30a, miR-30b, miR-30c-1, miR-30c-2, miR-30d, and miR-30e) and is encoded by six genes located on chromosome 1, 6, and 8 [18]. miRNA-30 is generated from the cleavage of primary miRNAs (pri-miRNAs) and precursor miRNAs (pre-miRNAs) by Drosha and Dicer cleavage. MiRNA-30 -5p be generated from 5p arm of pre-miR − 30 [19]. The mature miR − 30 family share a common seed sequence near the 5′ end but possess different compensatory sequences near the 3′ end. These different compensatory sequences allow miR-30 family members to target different genes and pathways, thus performing corresponding biological function [18]. Several studies have indicated that miRNA-30 family plays a key role in regulation of cell proliferation and differentiation and organs development as well as their diseases [20, 21]. In terms of differentiation, role of miR-30 is controversial. For example, Guess et al., reported that up-regulation of miR-30 family members in myoblasts promotes differentiation [22]. Alternatively, down- regulation of miR-30 in an osteoblast precursor cell line induces differentiation [23]. In addition, high expression of miR-30 is reported in the mouse and human testis tissue, which is critical for male fertility and reproductive development [24, 25]. Further research is required to clarify signaling pathways of the miR-30 family in regulation of spermatogenesis.The purpose of this article was to investigate the effects of miRNA- 30a-5p in the differentiation process of neonatal mouse spermatogonial stem cells.

## Materials and methods

### Animals

Male BALB/c mice (3–6 days old) were purchased from the Faculty of Pharmacy, Tehran University of Medical Sciences. All in vivo studies were performed according to standard operational procedures and regulations provided by the Ethics Committee of Tehran University of Medical Sciences (IR.TUMS.MEDICINE.REC.1396.3940).

### Isolation of SSCs

SSCs were isolated according to the method proposed by Kanatsu-Shinohara with a slight modification [26]. First, anesthesia was done using 0.05 mg/kg ketamine (Sigma-Aldrich, St. Louis, MO); then, the removed testes were immediately transferred to dishes containing phosphate‐buffered saline (PBS; Sigma‐Aldrich). Under sterile conditions, PBS supplemented with 1 % pen/strep was used for washing the samples. In the first step of enzymatic digestion, the minced testes were transferred to a digestion medium containing 5 µg/mL DNase (Sigma‐Aldrich), 1 mg/mL collagenase type IV(Gibco,CA), and 1 mg/mL hyaluronidase (Sigma‐Aldrich) and then incubated for 20 min at 37 °C with 5 % CO2. The suspension was pipetted gently every 5 min. It was centrifuged at 1,500 g for 5 min. In the second step, the same digestion medium was used to purify cellular pellets for 15 min. Finally, the viability of the cells was evaluated with a hemocytometer using 0.04 % trypan blue.

### Enrichment of SSCs

Initially, for SSC enrichment, somatic cells were separated by differential plating to assess of the purity of SSCs [27–30]. Flow cytometry was carried out with ID4 and Thy1 markers. Briefly, 10^5^ cells were incubated in 100 µl PBS/FBS and 10 µl primary antibody for 1 h at 4 °C. The primary antibodies for identification of specific markers ID4 and Thy1 included ID4 (GTX89728, Geno Tex) and Thy1 (Lot 5,150,211,128), respectively. After washing twice with PBS, the cells were incubated in 100 µl PBS/FBS and 10 µl secondary antibody for 1 h at 4 °C. FITC goat anti-rabbit IgG H&L (ab6717, Abcam, UK) was used as the secondary antibody for ID4 and Thy1. The cells considered as control cells were not incubated with any antibodies. Finally, the cells were kept in a dark room on ice and the purity percentage was determined by flow cytometry.

### Culture of SSCs

To culture purified cells (2 × 10^5^ cell/cm^2^), a medium containing Dulbecco’s modified Eagle’s medium (DMEM) supplemented with 10 % fetal bovine serum (FBS, Life Technologies), 10 ng/Ml Leukemia Inhibitory Factor (LIF; Sigma, Haverhill), 10 ng/mL basic fibroblast growth factor (Peprotech, Rocky Hill, NJ), 0.1 mM β-mercaptoethanol (Sigma‐Aldrich), 10 ng/mL glial cell line‐derived neurotrophic factor (GDNF; Sigma‐ Aldrich), 100 U/mL penicillin (Sigma‐Aldrich, Darmstadt), and 100 µg/mL streptomycin (Sigma, Germany) was used. All cultures were incubated at 37 °C in a humidified 5 % CO incubator and the medium was refreshed every 2–3 days.

### Alkaline phosphatase staining

The Fast Red TR/Naphthol AS-MX tablets (Sigma USA, F4648) were used to evaluate alkaline phosphatase activity. Alkaline dye was produced by adding Tris buffer to Naphthol tablets. The cells were then incubated with the dye for 30 min at room temperature and observed under an inverted microscope (IX71, Olympus, Japan).

### Transfection of miR-30a-5p inhibitor into Mouse SSCs

MiRNA-30a-5p Inhibitor (Sigma- MLTUD0016) was provided from Sigma-Aldrich. Dosimetry experiments were done for optimization of lipofectamine 2000 and miR-30a- 5p inhibitor concentration, respectively. Eventually, 100 nM and 0.3 nM were determined as optimal doses for miR-30a- 5p inhibitor and lipofectamine, respectively. The cultured SSCs were divided into four groups: (1) no miRNA − 30a transfection, (2) miRNA-30a- 5p inhibitor, (3) miRNA-30a- 5p inhibitor control, (4) lipofectamine alone.

In this study, lipofectamine 2000 transfection agent was used for transfection of miRNA-30a- 5p inhibitor into SSCs. First, miR-30a- 5p inhibitor was diluted in 125 µl Opti-MEM (Invitrogen) reduced serum medium (Cat. No. 31985-062). Then, the mixed medium was incubated for 5 min at room temperature (RT). At the same time, 1 µl Lipofectamine 2000 transfection agent (Invitrogen) was added to 50 µl Opti-MEM reduced serum medium and incubated for 5 min at RT. Furthermore, diluted miR-30a- 5p inhibitor and lipofectamine were incubated for 20 min at RT. Finally, the transfection medium was added directly to the freshly seeded cells (0.4–1.6 × 10^5^ of SSCs in a 24-well plate) [31]. The transfection medium was replaced 4 h later by fresh growth medium and the samples were incubated at 37 °C in 5 % CO2 for 48 h. After 48 h of transfection, the cells were collected for evaluation of changes in the expression of proteins. Colonization of SSCs was assessed after one week.

### Detection of miR-30a-5p expression in SSCs before and after transfection

To verify the effectiveness of miR-30a-5p transfection in SSCs, the amount of miR-30a-5p was measured before (intracellular miR-30a-5p) and 48 h after the transfection using the quantitative real-time PCR technique. In fact, the intracellular miR-30a-5p expression was measured before the transfection and compared with the level of miR-30a-5p expression after the transfection of miR-30a-5p inhibitor.

TRIzol reagent (Invitrogen, USA) was used for complete RNA extraction. Then, cDNA was synthesized using the Reverse Transcription System Kit (ZistRoyesh, Iran) according to the manufacturer’s instruction. Quantitative real-time PCR was performed with GoTaq qPCR Master Mix (Promega) using the ABI7500 (Applied Biosystems, Foster City, California, USA). Melting curve analysis was used to identify the non-specific real-time PCR products. U6 snRNA was selected as an internal control gene and the miR-30a-5p expression was calculated through 2-∆∆CT method [31, 32]. Quantitative real-time PCR was done in triplicate for each sample. The sequence of designed primers were as follows:

- Forward primer (miR 30a-5p): GCGTGTAAACATCCTCGAC.
- Reverse primer (miR 30a-5p): GTGCAGGGTCCGAGGT.
- Forward primer (U6): CTCGCTTCGGCAGCACA.
- Reverse primer (U6): AACGCTTCACGAATTTGCGT..

### Assessment of SSCs colonization

After transfection, 2 × 105 cell/cm2 SSCs were seeded and cultivated in a DMEM containing 10 % FBS, 100 U/mL penicillin, 100 µg/mL streptomycin, 10 ng/ml leukemia inhibitory factor, and 10 µg/mL glial cell line-derived neurotrophic factor in 5 % CO2 at 37 °C for one week. The diameter and number of colonies were determined using an inverted microscope (Olympus‐ CKX41‐ JAPAN). Subsequently, the data were analyzed using the Image J software (version 1.240; National Institutes of Health, Bethesda, MD, USA) [33].

### Assessment of c-Kit and STRA8 expression by immunocytochemistry

Immunocytochemistry was done to assess the factors related to SSCs differentiation. At the first, SSCs were fixed with paraformaldehyde 4 % (Sigma-Aldrich) and permeabilized with 0.1 % Triton X-100 (Sigma-Al-drich) followed by blocking for one hour with 10 % goat serum (Sigma-Aldrich). Then, Samples were incubated with c-Kit (ab5506, Abcam, UK), and STRA8 (ab15093, Abcam, UK) for 24 h, followed by 2-hour exposure with secondary antibody fluorescein isothio-cyanate (FITC; ab6717, Abcam, UK). In addition, nuclei were stained with 4¢, 6-diamid-ino-2-phenylindole (DAPI, 1 lg/mL). Finally, the slides were observed by a fluorescence microscope (Olympus LX71, Japan).

### Evaluation of GFRA1, PLZF and ID4 expression by western blot

TriPure Isolation Reagent (Roche, Germany) was used to extract total protein from SSCs. Electrophoresis was done to separate 20 µg of the total protein for each sample. Then, the proteins were transferred into 10.5 and 12.5 % gradient sodium dodecyl sulfate (SDS)-polyacrylamide gel (BioRad Laboratories, Hercules, CA) and polyvinylidene difluoride (PVDF) membranes (Roche, Germany) and blocked with 5 % non‐fat dry milk (Carnation, CA). Subsequently, the samples were incubated with primary antibodies against GFRα1 (1:1000), PLZF (1:1000) and ID4 (1:1000) overnight at 4 °C. Nitrocellulose membrane was incubated for 2 h after adding appropriate secondary antibodies (HRP conjugated goat anti‐rabbit) (Abcam). Finally, the expression of the proteins was evaluated using enhanced chemiluminescence.

### Statistical analysis

The data were analyzed with GraphPad Prism 7.0 (GraphPad Software, Inc., La Jolla, California) using one-way ANOVA and Tukey post-hoc test. The statistical significance was set at 0.05 (*P* ≤ 0.05). The data are presented as the mean ± standard deviation (SD).

## Results

### Determining purification of SSCs

**Fig. 1:**
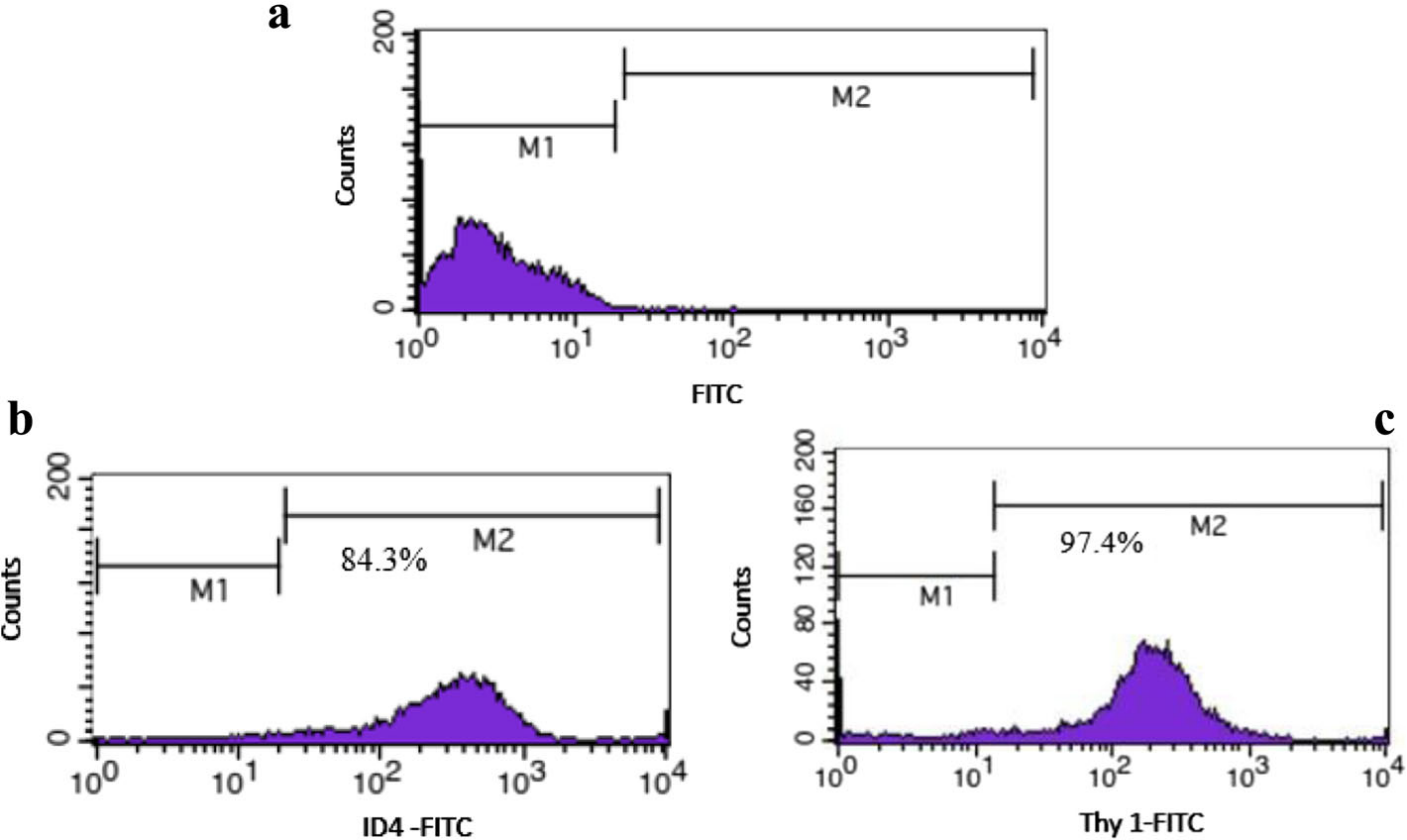
Results of flow cytometry indicating the purity percentage of SSCs with ID4 and Thy1 markers. M1: marker negative cells, M2: marker positive cells. **a**: Control; **b**: Thy1 positive **c**: ID4 positive

Expression of undifferentiated spermatogonial markers ID4 and Thy1 for identification of SSCs was investigated using flow cytometry. As expected, the percentage of the expression of markers was 84.3 and 97.4 % in the SSCs (Fig. 1).

### Assessment of miR-30a-5p expression in SSCs

**Fig. 2:**
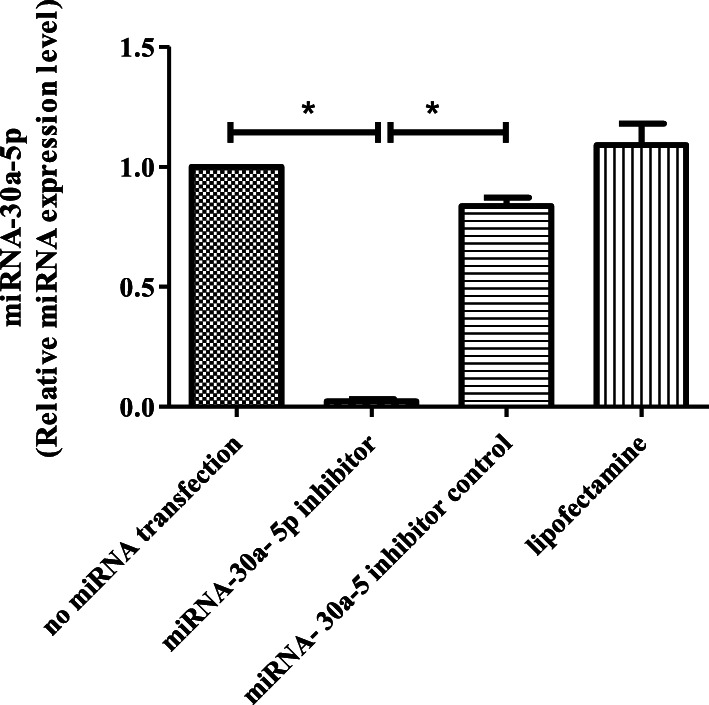
Transfection efficiency of miRNA-30a-5p inhibitor in SSCs. miRNA-30a-5p expression reduced significantly in the miRNA-30a-5p inhibitor group. Experiment was performed in triplicate. There were 2 × 10^5^ cell/cm2 of SSCs in each group. The results are reported as mean ± SD. ***p < 0.001

MiR-30a-5p expression was evaluated before and 48 h after transfection by using the quantitative real-time PCR technique. The results indicated that the expression level of miR-30a-5p significantly decreased in the miR-30a-5p inhibitor group (22.51 ± 3.51) compared to the untransfected group (*p* ≤ 0.001). No significant changes were observed in the inhibitor control and lipofectamine groups (Fig. 2).

### SSC morphology and confirmation of colonies

**Fig. 3:**
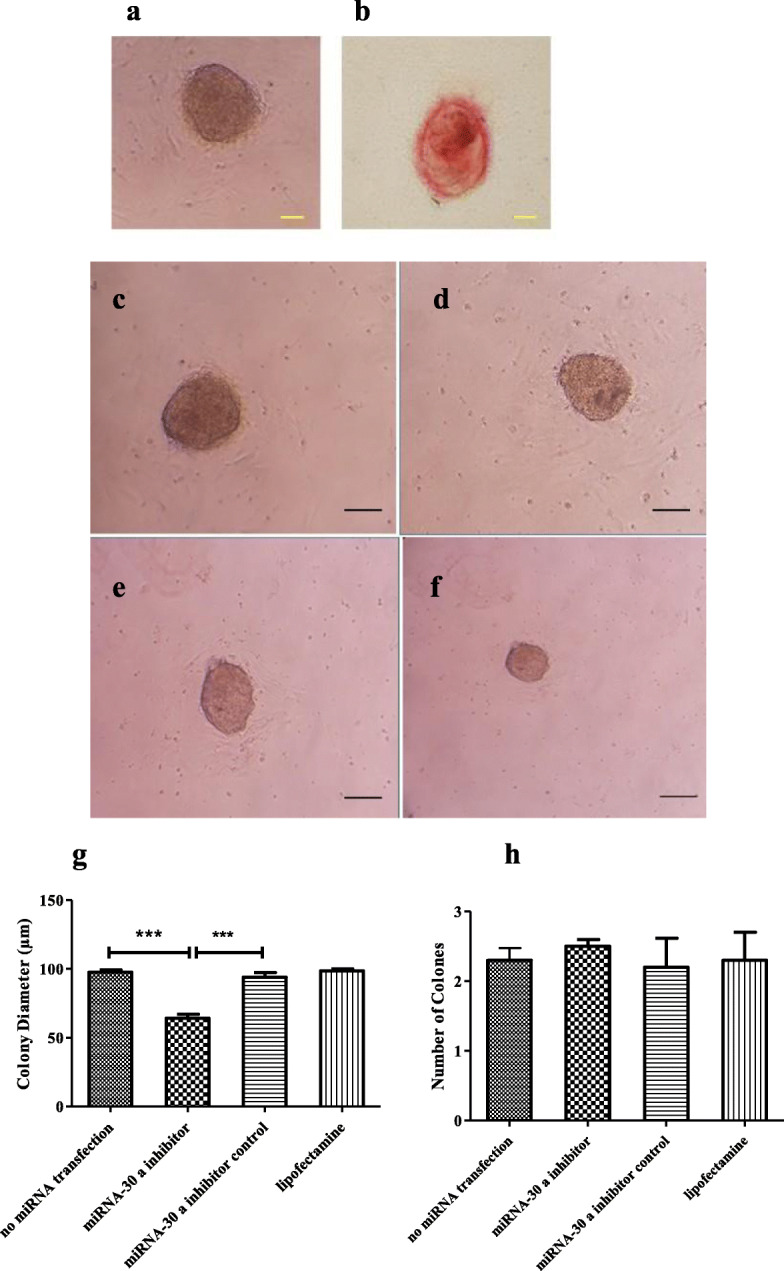
Assessment of SSCs colonization after transfection. **a, b**: Verification of colonies with alkaline phosphatase staining after culturing. A: colony of SSCs. B: Alkaline phosphatase positive colony Scale bars = 100 μm. **c, d, e, f**: Microscopic morphology of SSC colonies. **c**: no miRNA transfection group; D: miRNA-30a-5p inhibitor control Group. E: lipofectamine group. F: miRNA-30a-5p inhibitor group. **g, h**: Comparison of the diameter (**g**) and (**h**) number of SSC colonies between study groups. Experiments were performed in triplicate. There were 2 × 10^5^ cell/cm2 of SSCs in each group.The results are reported as mean ± SD ***p < 0.001

After the purification process, SSCs were cultured to reach optimal confluence before transfection. Alkaline phosphatase staining was done to confirm SSC colonies. The red color of the colonies indicated the stem cell alkaline phosphatase activity in the colonies (Fig. 3a,b).

### Colony assay

One week after transfection, the images of the colonies were captured by an inverted microscope and the diameter and number of colonies were measured using the Image J software (Fig. 3c-f). The diameter of the colonies was significantly smaller in the miRNA-30a-5p inhibitor group (195.9 ± 6.06 μm) compared to other group whereas no significant changes were observed in the number of colonies (4.63 ± 0.37) between miRNA-30a-5p inhibitor and other groups (Fig. 3g,h).

### Immunocytochemistry findings

**Fig. 4:**
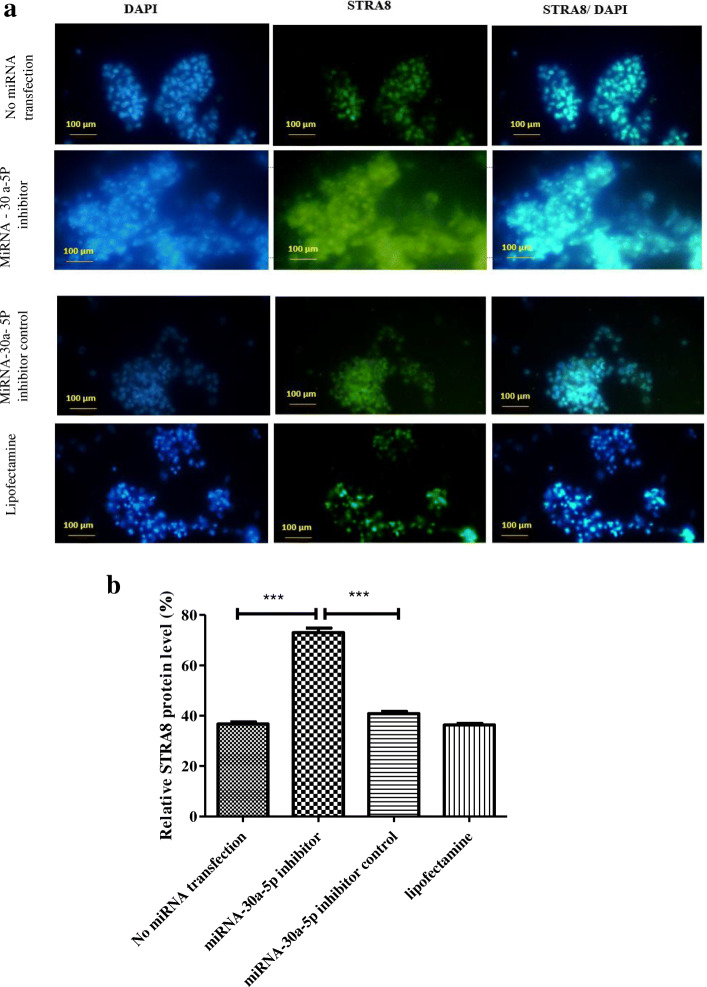
Immunocytochemistry for identifying STRA8 differentiation marker 48 h after transfection. **a**: SSCs were immunostained with antibody for STRA8 marker (green). DAPI was used for nuclear staining (blue). The images showed that the expression of STRA8 protein increased significantly in the inhibitor group compared to other groups. (Scale bars = 100 μm). **b**: Graph is indicative of the quantified area with fluorescent staining for STRA8 marker. Experiments were performed in triplicate. There were 2 × 10^5^ cell/cm2 of SSCs in each group. The results are reported as mean ± SD ***p < 0.001

**Fig. 5:**
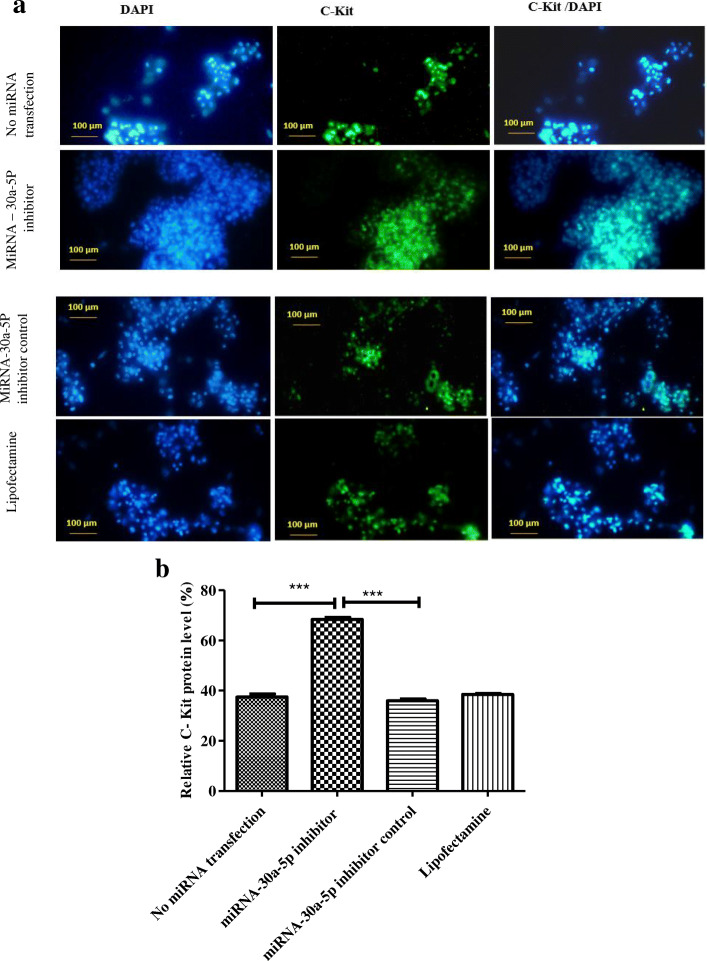
Immunocytochemistry for identifying C-Kit differentiation marker 48 h after transfection. **a**: SSCs were immunostained with antibody for C-Kit marker (green). DAPI was used for nuclear staining (blue). The images showed that the expression of C-Kit protein increased significantly in the inhibitor group compared to other groups. (Scale bars = 100 μm). **b**: Graph is indicative of the quantified area with fluorescent staining for C-Kit marker. Experiments were performed in triplicate. There were 2 × 10^5^ cell/cm2 of SSCs in each group.The results are reported as mean ± SD ***p < 0.001

Stra8 and c-Kit as markers of differentiation were evaluated 48 h after transfection using immunocytochemistry. The percentage of expression for stra8 showed a marked increase in SSCs transfected with miR-30a-5p inhibitor (72.93 ± 3.21) compared to other cells (Fig. 4). Similarly, the expression of c-Kit differentiation gene also increased significantly in the inhibitor group (68.37 ± 1.38) compared to other groups (Fig. 5). Collectively, the results indicated that inhibition of miR-30a-5p induced the differentiation process in mouse SSCs.

### Western blot findings

**Fig. 6:**
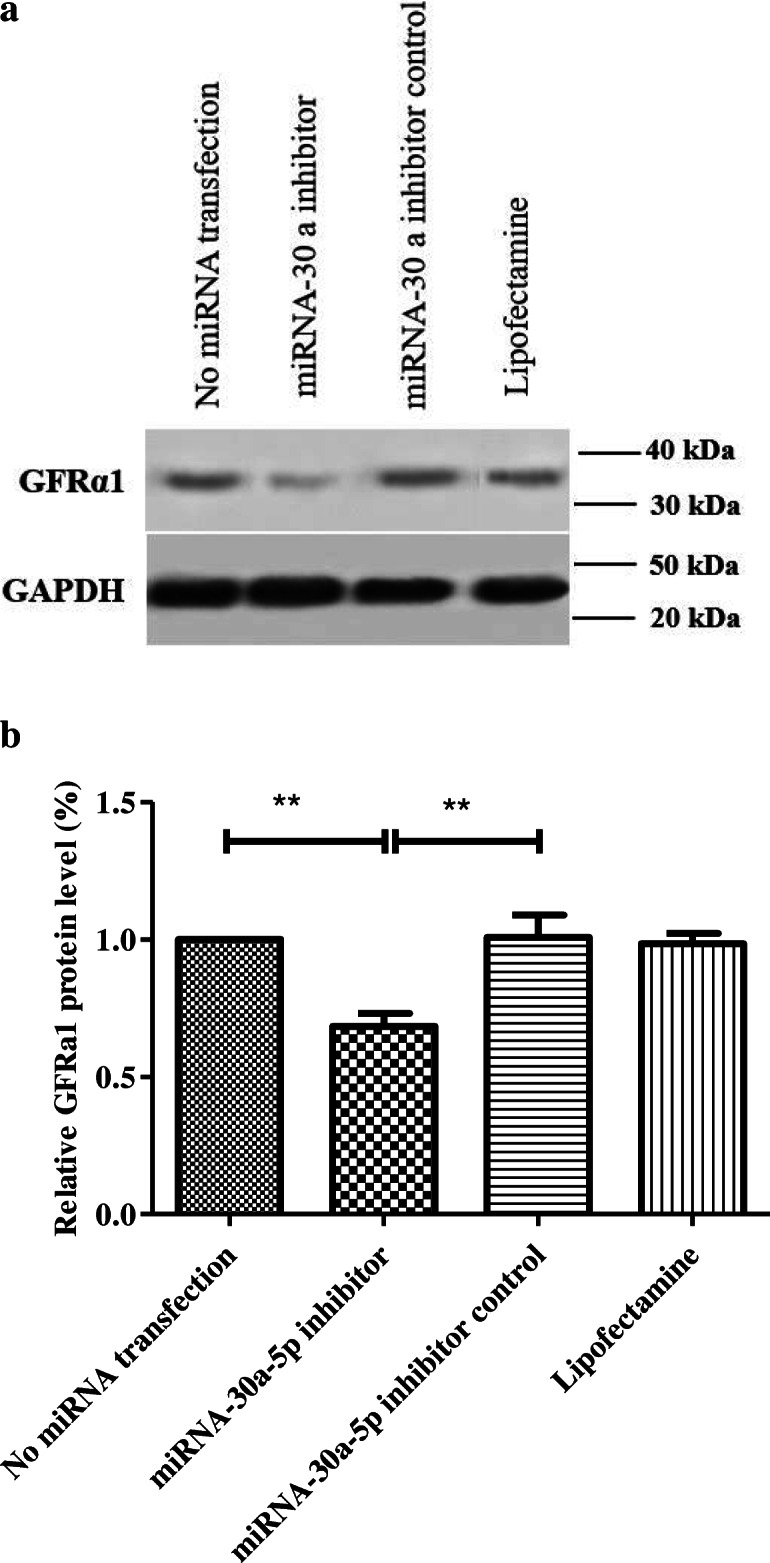
Western analysis for assessment of GFRa1 48 h after transfection in different groups. **a**: GAPDH was used as the internal control; **b**: Graph presents the ratio for normalization of the density of the marker to the GAPDH. Experiments were performed in triplicate. There were 2 × 10^5^ cell/cm2 of SSCs in each group .The results are reported as mean ± SD ***p < 0.001

**Fig. 7:**
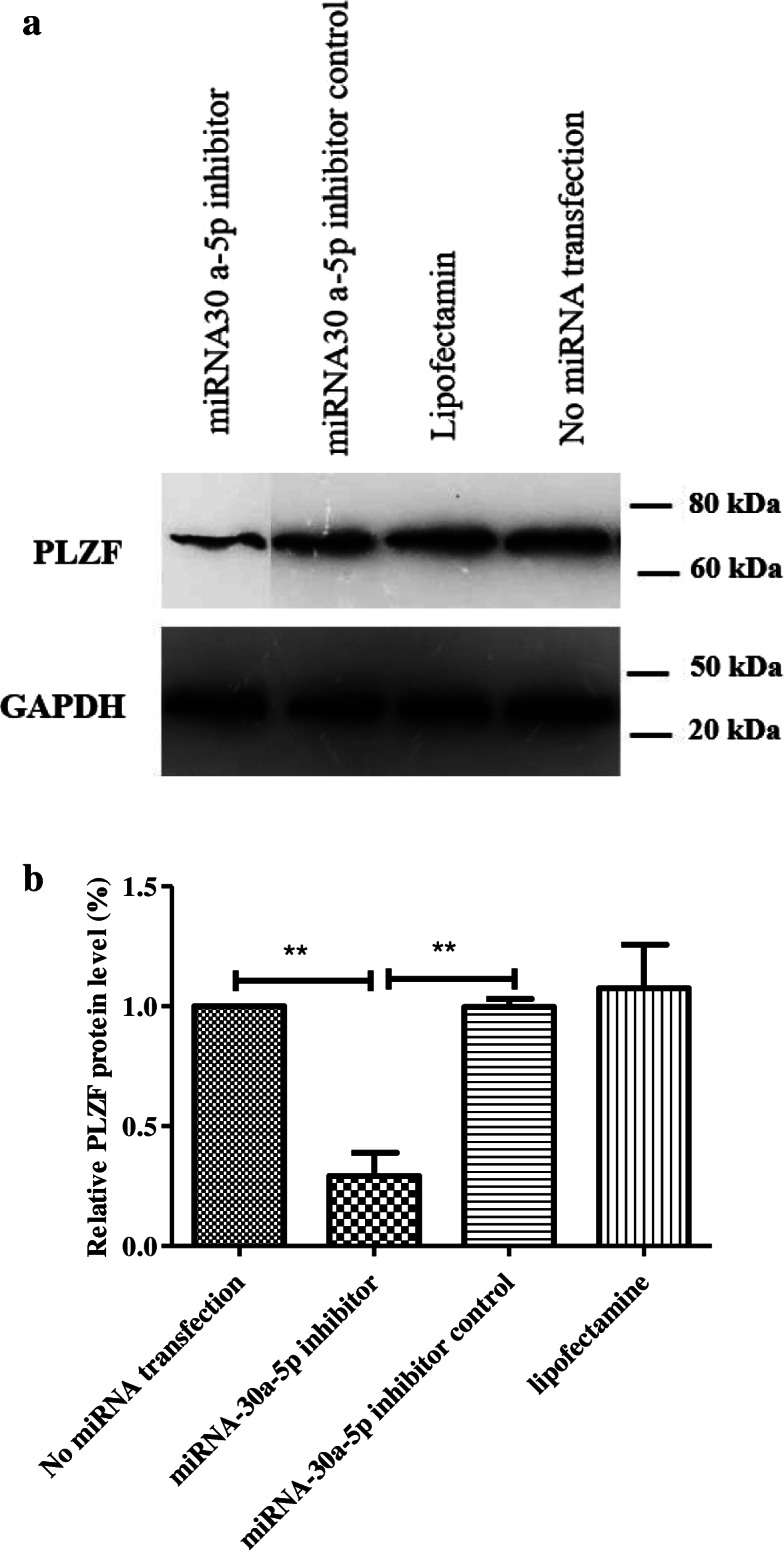
Western analysis for assessment of PLZF 48 h after transfection in different groups.** a**: GAPDH was used as the internal control; **b**: Graph presents the ratio for normalization of the density of the marker to the GAPDH. Experiments were performed in triplicate. There were 2 × 10^5^ cell/cm2 of SSCs in each group. The results are reported as mean ± SD ***p < 0.001

**Fig. 8:**
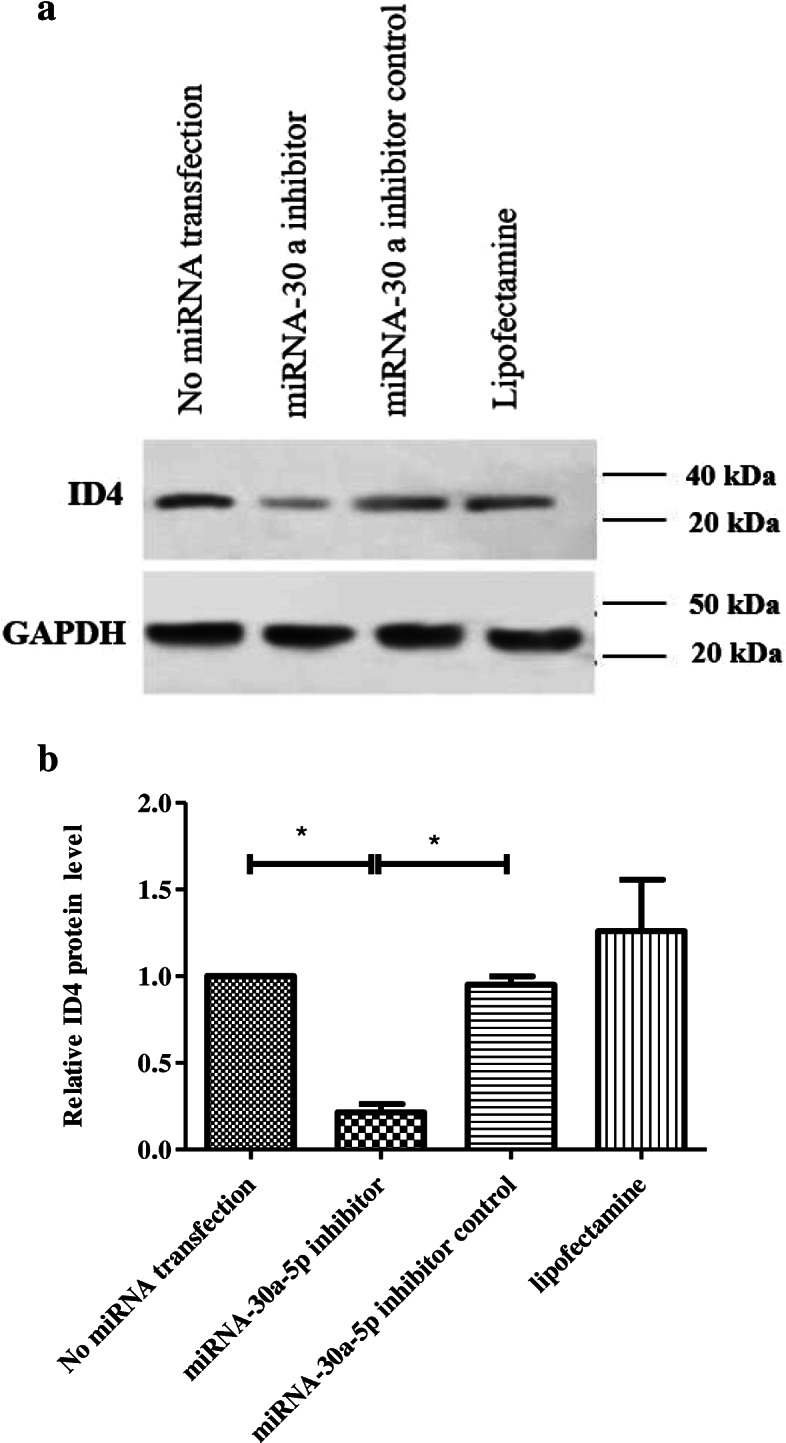
Western analysis for assessment of ID4 48 h after transfection in different groups. **a**: GAPDH was used as the internal control; **b**: Graph presents the ratio for normalization of the density of the marker to the GAPDH. Experiments were performed in triplicate. There were 2 × 10^5^ cell/cm2 of SSCs in each group. The results are reported as mean ± SD ***p < 0.001

Western blot was used to investigate the expression of proteins related to proliferation (GFRa1, PLZF and ID4) 48 h after transfection. According to the results, there was a marked reduction in the expression of GFRa1 in miR-30a-5p group (0.65 ± 0.08) compared to other groups (Fig. 6). The expression of PLZF proliferation protein decreased in SSCs transfected with miR-30a-5p inhibitor (0.29 ± 0.16) compared to other cells (Fig. 7). Analysis of the result of ID4 protein also showed a notable reduction in the inhibitor group (0.2145 ± 0.08) versus other groups (Fig. 8). In fact, downregulation of miR-30a inhibited SSCs self-renewal and proliferation.

## Discussion

Spermatogonial stem cells have a fundamental role in supporting and promoting spermatogenesis. Spermatogenesis is a highly organized procedure that needs balance between proliferation and differentiation of SSCs. This balance is regulated by intrinsic factors and extrinsic signals and plays a pivotal role in determination of lifelong supply of spermatozoa and fertility [34, 35]. In this regard, understanding of intrinsic regulators and their roles in regulating the fate of SSCs is necessary as preclinical studies have shown that MiRNAs are critical for biological processes and act via binding to the 3′ untranslated region (3′UTR) of their specific mRNAs. Recent studies have revealed that a wide range of miRNAs may contribute to spermatogenesis in mammals via regulation of target genes expression [10, 36]. However, molecular mechanisms and targets of individual miRNAs in regulation of spermatogenesis are mostly unknown. In the present study, we investigated the role of miR-30a-5p in the fate of SSCs after exposure to miR − 30a-5p inhibitor.

After enzymatic digestion and differential platting, the SSCs were identified by ID4 and Thy1 surface markers. Several studies have reported that ID4 expression is limited to A_s_ spermatogonia and overexpression of ID4 inhibits the stem-to-progenitor transition [37, 38]. In fact, ID4 plays a key role in the regulation of SSCs’ self-renewal or undifferentiated state. Therefore, ID4 can be used for identification of SSCs.

Thy-1 is a glycosyl phosphatidylinositol-anchored glycoprotein of the Ig superfamily that has been introduced as a confirmed marker of SSCs in bovine, rodents, and primates [39]. Many researchers also enriched SSCs based on Thy-1 and achieved a high purity of SSCs. A considerable point in this regard is that although these markers have been introduced by other researchers as possible markers for SSC identity, no exclusive marker has yet been recommended for purification of SSCs. Other markers such as Plzf, α6, β1, and GFR α1 have also been used for purification of SSCs in the literature [31, 32, 38]. In this study, the high percentage of expression for these markers confirmed that the collected cells were undifferentiated since SSCs first appear at 3–6 days postpartum in mice. Furthermore, the ability of cultured cells for self-renewal and colony formation also confirmed that the cells were undifferentiated. These results were consistent with other studies [4, 6, 14]. According to the literature, Sertoli cells as the only somatic cells in the seminiferous tubules play vital roles in the regulation of the balance between SSCs self-renewal and differentiation by secreting many growth factors [40]. Recent studies have also shown that some miRNAs have a key role in proliferation, maturation and hormone responses of Sertoli cell [41, 42]. Considering the roles of Sertoli cells in SSCs fate determination and recent discoveries regarding the regulatory role of miRNA in Sertoli cells and androgen-dependent spermatogenic events, differential plating was used in the present study to separate SSCs from Sertoli cells and somatic cells to evaluate the effect of miRNA on the SSCs fate. Similarly, some other studies used differential plating to evaluate the effects of miR − 34c, miR- 20 and miR- 106a on spermatogenesis [6, 16]. Several studies have shown that GFRα1 expression is dominantly detected in mouse undifferentiated SSCs. GFRα1 is the main receptor for GDNF growth factor and is considered a necessary portion of the GFRα1/RET complex [43, 44]. In other words, GDNF needs GFRα1 for induction of RET autophosphorylation and RET should be co-expressed with the GFRα1 receptor to bind to GDNF. GDNF, as an indispensable factor for maintenance of SSCs, acts via binding to the GFRα1/RET receptor and regulates the expression of genes involved in promotion of SSCs self-renewal or prevention of SSCs differentiation, including ID4, Nanos2, Etv5, and Bcl6b. Recent studies have shown that downregulation of Gfra1 leads to inactivation of RET tyrosine kinase, which may sequentially block the intracellular GDNF/GFRA1/ RET signaling pathway [43, 44]. In fact, this molecular mechanism leads to inhibition of the proliferation and entry of SSCs into the differentiation process. Furthermore, GDNF alone is unable to achieve this goal and needs to collaborate with GDNF independent proteins including PLZF, FOXO1, GILZ and TAF4B. Oatley et al. found that ID4 was a maintenance factor for SSCs and ID4 was induced by GDNF in Thy1^+^ spermatogonial cell cultures [3].

Although the exact mechanism of PLZF in SSCs self-renewal has not been elucidated, several studies have suggested different mechanisms for promoting mouse SSCs self-renewal. PLZF indirectly inhibits the mTORC1 pathway through activation of Redd1. In other words, depletion of PLZF leads to mTORC1 activation, downregulation of GDNF receptors (including GFRa1 and c-Ret), suppression of the SSCs response to GDNF, and repression of SSCs proliferation. In addition, PLZF also directly and indirectly inhibits the expression of c-kit (differentiation gene) [45, 46, 47].

We evaluated the expression of GFRα1, PLZF and ID4 proteins 48 h after transfection using the western blot method and the results showed downregulation of the expression of GFRα1, PLZF and ID4 proteins. Therefore, we believe that downregulation of miR-30a- 5p reduced SSCs self-renewal and proliferation. Although its exact mechanism is not fully clear, it is suggested that downregulation of miR-30a- 5p probably leads to reduction of PLZF and activation of mTORC1, and reduction of GFRA1. Depletion of Gfra1 also results in RET inactivation, blockade of the GDNF/GFRA1/ RET signaling pathway, and reduction of the ID4 expression as a gene involved in promotion of SSCs self-renewal. In other words, it can be concluded that miR-30a-5p inhibitor suppresses the SSCs response to adding GDNF in culture medium and may reduce SSCs proliferation and self-renewal through this molecular mechanism.

C-kit is a member of the class III receptor tyrosine kinases, which is only found in differentiated spermatogonia and is considered a well-characterized marker of spermatogonial differentiation. The transition of SSCs from an undifferentiated state to a differentiated state coincides with activation of the Kit/Kitl system [48]. Followed by c-kit activation, differentiated SSCs will enter meiosis and start to express early meiotic markers including STRA8, Dmc1 and Scp3 [47]. The molecular mechanisms regulating c-kit expression in spermatogenesis steps are not very clear. However, several studies have discovered that PLZF represses c-Kit transcription.

The results of ICC showed that the expression of differentiation proteins (c-Kit and STRA8) increased significantly in SSCs transfected with miR-30a-5p inhibitor compared to other groups. These results indicated that inhibition of miR-30a- 5p in SSCs causes the cells to enter the differentiation stage and induces meiosis in them. As a possible mechanism involved in this process, it can be suggested that downregulation of miR-30a- 5p leads to reduction of PLZF and activation of c-kit. In this way, the expression of STRA8 increases, indicating the differentiation of the cells and the onset of meiosis.

In line with this study, Chen et al. found that miR-202-3p inhibitor induced differentiation in SSCs through reducing the expression of Plzf and increasing the expression of Stra8, Dazl and Sycp3 [15]. Wang et al. also found that transfection of miR-322 inhibitor led a significant decrease in Gfrα1, Etv5 and Plzf expression and a marked increase in the expression of C-kit, stra8 and Bcl6 in mouse SSCs [4].

On the other hand, it has been reported that up-regulation of miR-30 family members in myoblasts promotes differentiation [22]. Also inhibition of miR-30 family leads to a marked decrease in the proliferation and a major increase in the differentiation of intestinal epithelial cells [49].

According to our results and these studies can be suggested that effect of miR-30 in differentiation is cell type-specific depended. Indeed cell type-specific analyses on miRNA regulatory networks is important. Moreover, further research is warranted to evaluate miR-30 regulatory networks in stem cells fate.

## Conclusions

According to the results of this study, miR-30a-5p inhibitor induces differentiation in spermatogonial stem cells through downregulation of markers related to proliferation and upregulation of differentiation markers. The findings may be used for designing promising therapeutic strategies in infertility cases. Future studies should be conducted to clarify key signaling pathways in spermatogenesis since this knowledge would pave the way for designing more effective therapeutic strategies for infertility treatment.

## Supplementary Information

**Additional file 1.**

## Data Availability

All data generated or analyzed during this study are included in this published article.

## Abbreviations

SSCs: Spermatogonial Stem Cells
STRA8: Stimulated By Retinoic Acid 8
GDNF: Glial Cell-Derived Neurotrophic Factor
BMP4: Bone Morphogenetic Protein 4
PLZF: Promyelocytic Leukemia Zinc Finger
Stra8: Stimulated by retinoic acid 8
GFRα1: GDNF family receptor α1
KL: Kit ligand
LIF: Leukemia inhibitory factor
ICC: Immunocytochemistry
ID4: Inhibitor of DNA binding 4
