# Supplementary material for: MicroRNA-30a-5p promotes differentiation in neonatal mouse spermatogonial stem cells (SSCs)
Source: Reprod Biol Endocrinol. 2021 Jun 9;19:85. doi: 10.1186/s12958-021-00758-5 (PMC8188658; doi:10.1186/s12958-021-00758-5)

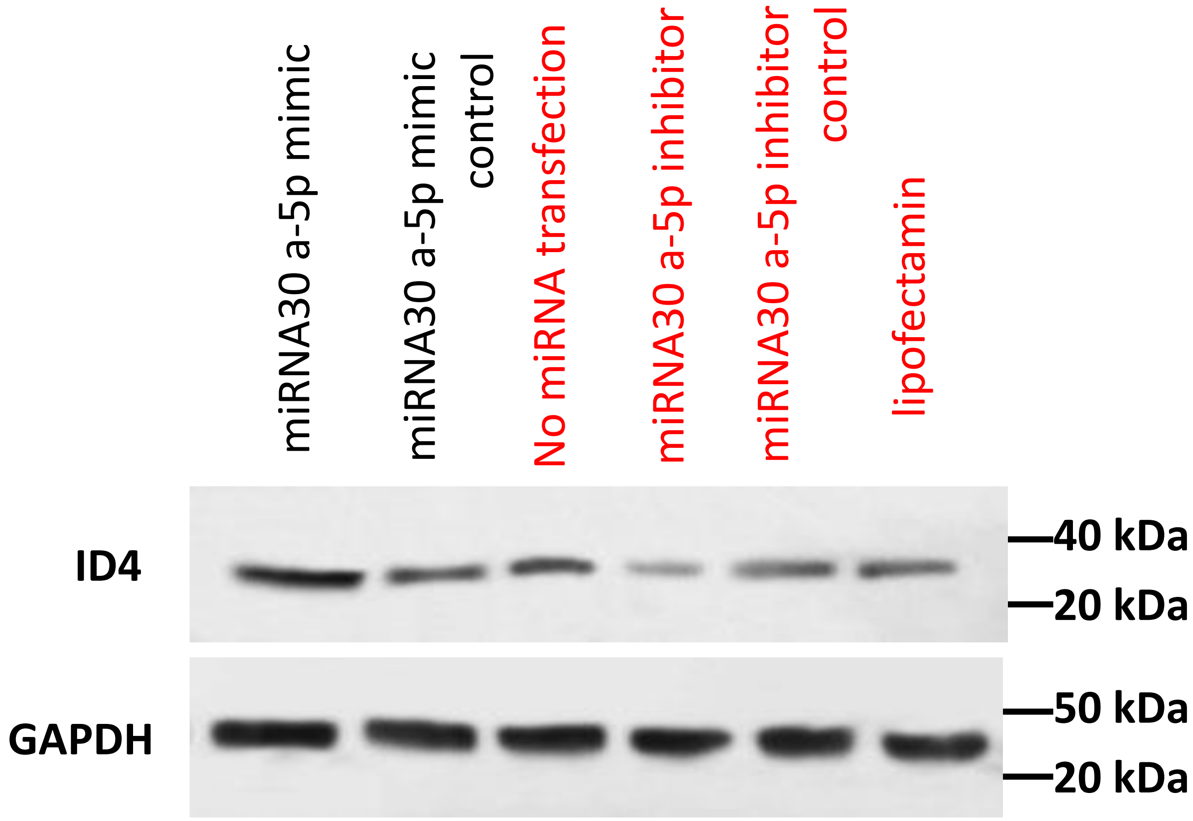
miRNA30 a-5p mimic
miRNA30 a-5p mimic control
No miRNA transfection
miRNA30 a-5p inhibitor
miRNA30 a-5p inhibitor control
lipofectamin
ID4
GAPDH
40 kDa
20 kDa
50 kDa
20 kDa

ID4

GAPDH

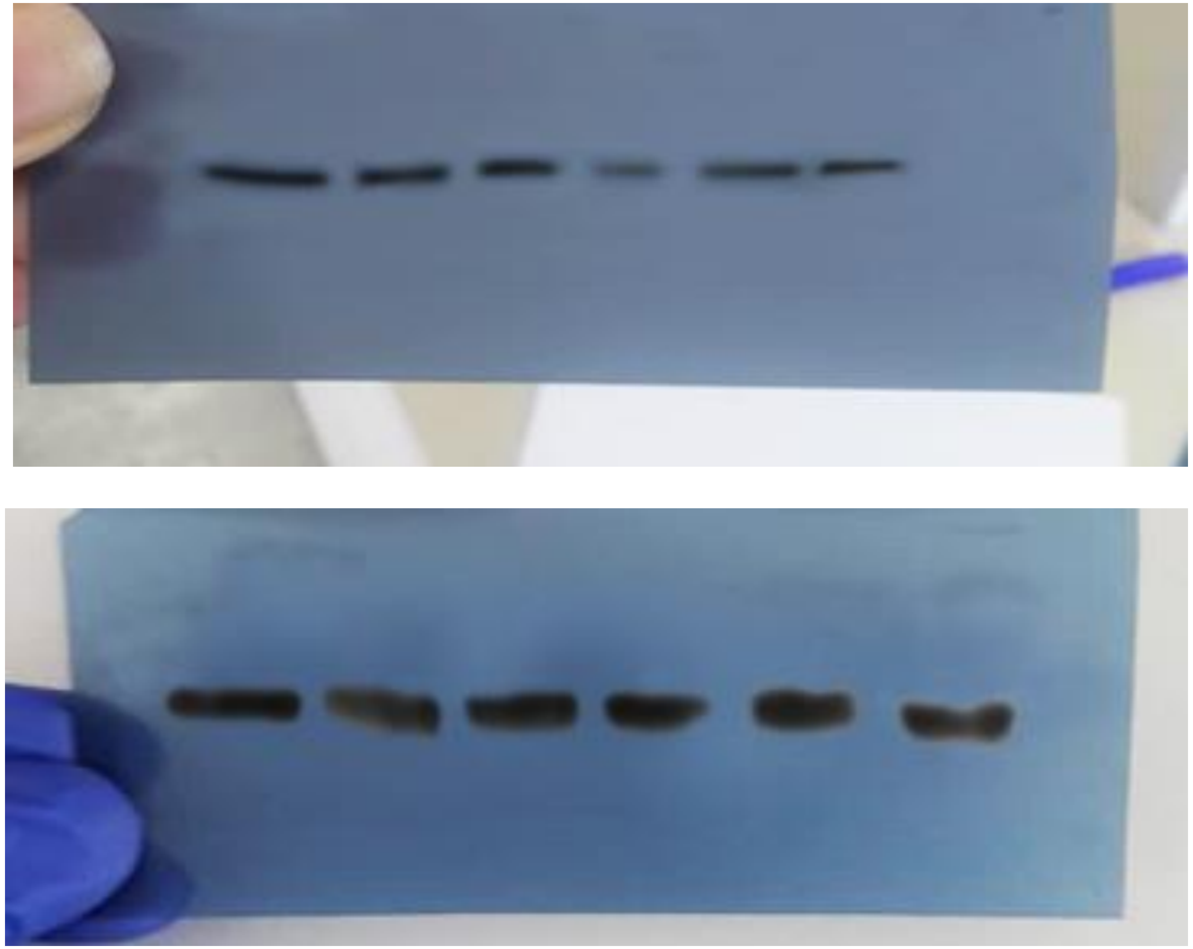

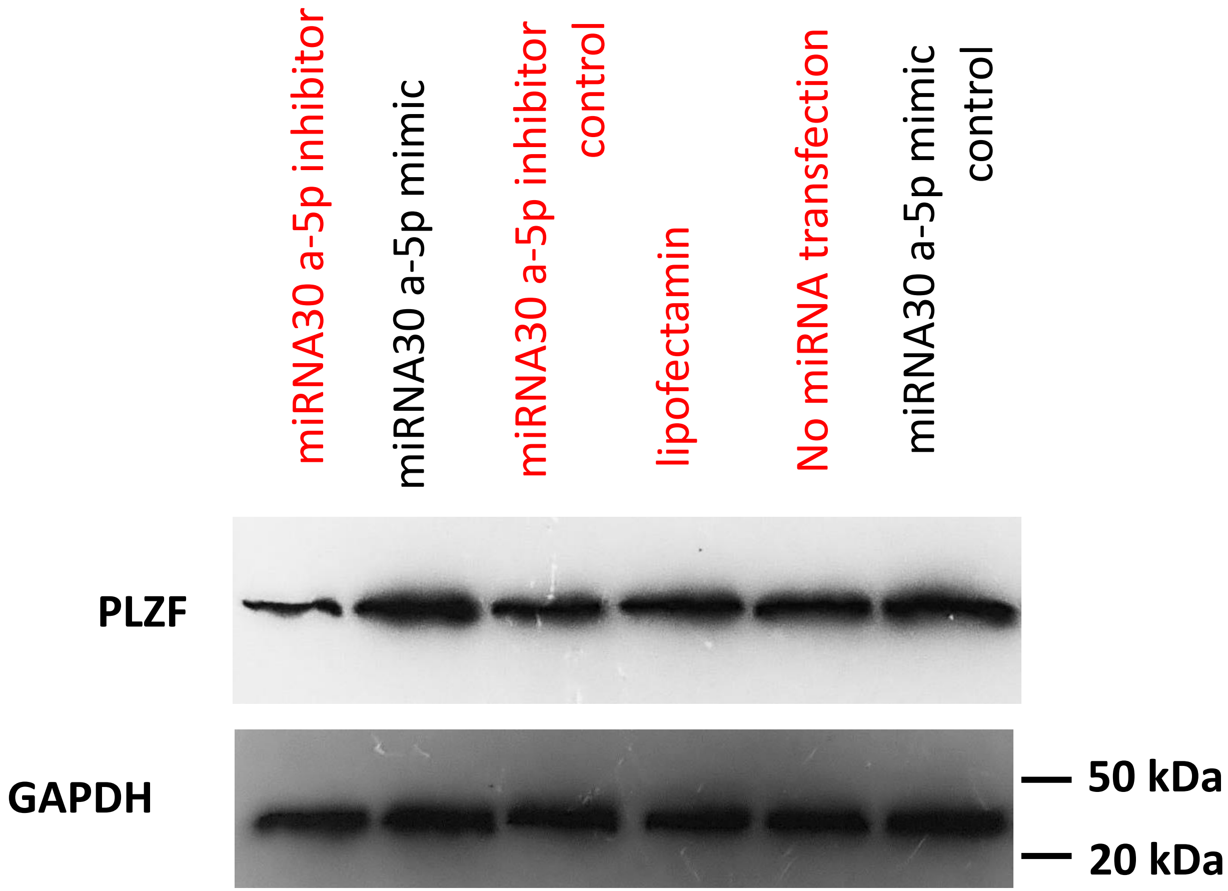
miRNA30 a-5p inhibitor
miRNA30 a-5p mimic
miRNA30 a-5p inhibitor control
lipofectamin
No miRNA transfection
miRNA30 a-5p mimic control
PLZF
GAPDH
50 kDa
20 kDa

PLZF

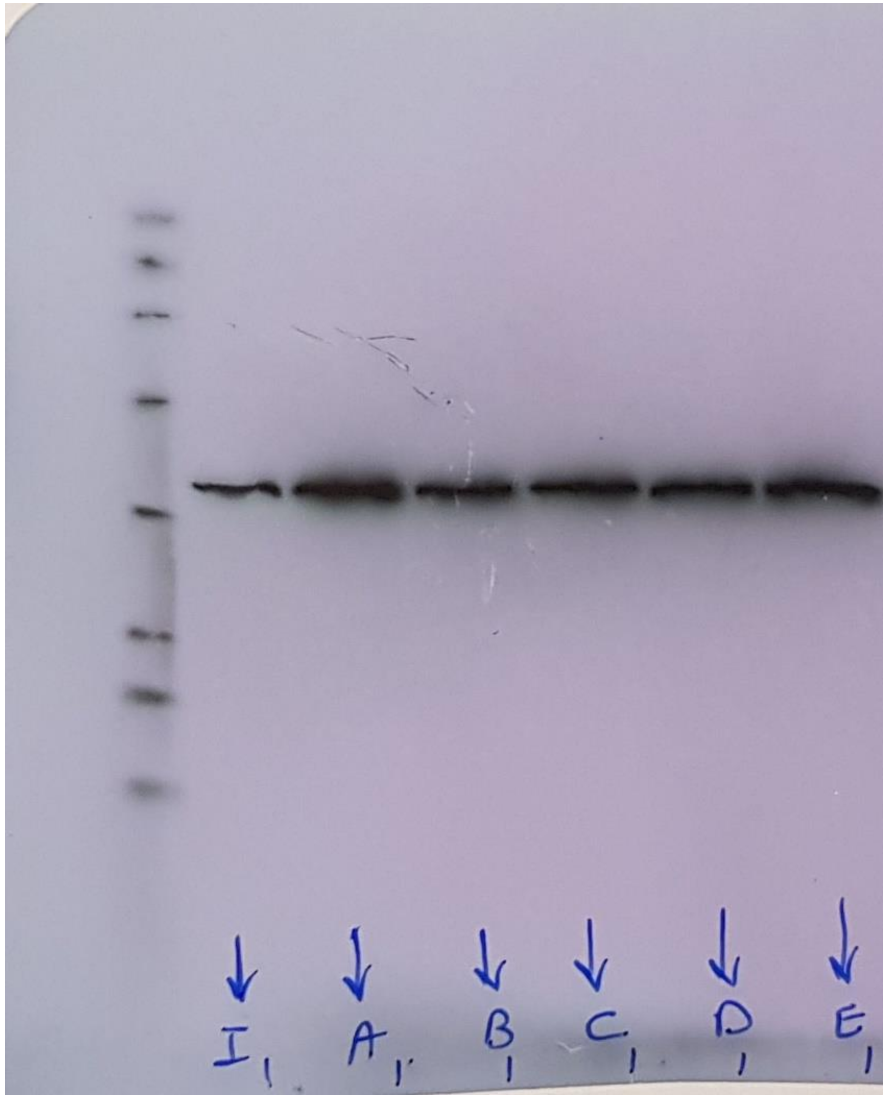

GAPDH

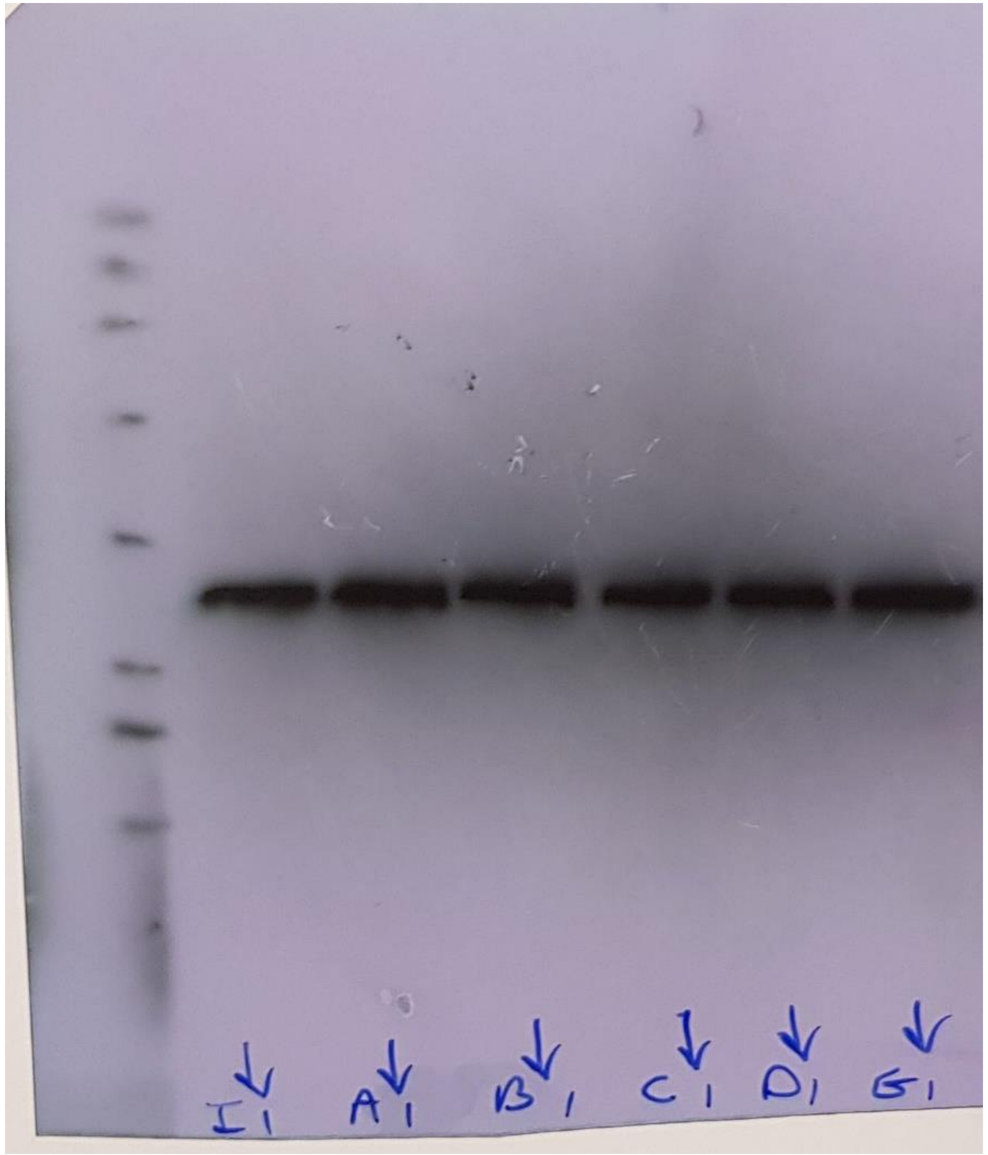

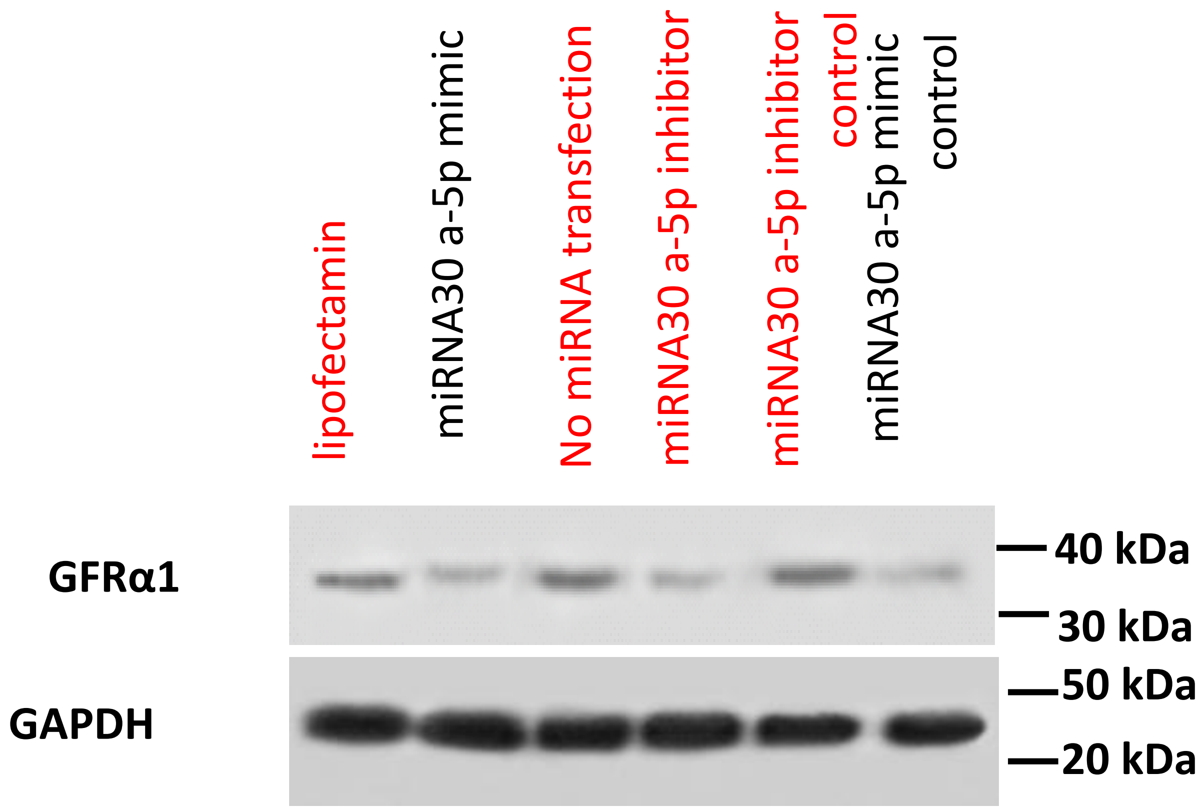
lipofectamin
miRNA30 a-5p mimic
No miRNA transfection
miRNA30 a-5p inhibitor
miRNA30 a-5p inhibitor control
miRNA30 a-5p mimic control
GFRα1
GAPDH
40 kDa
30 kDa
50 kDa
20 kDa

GFRα1

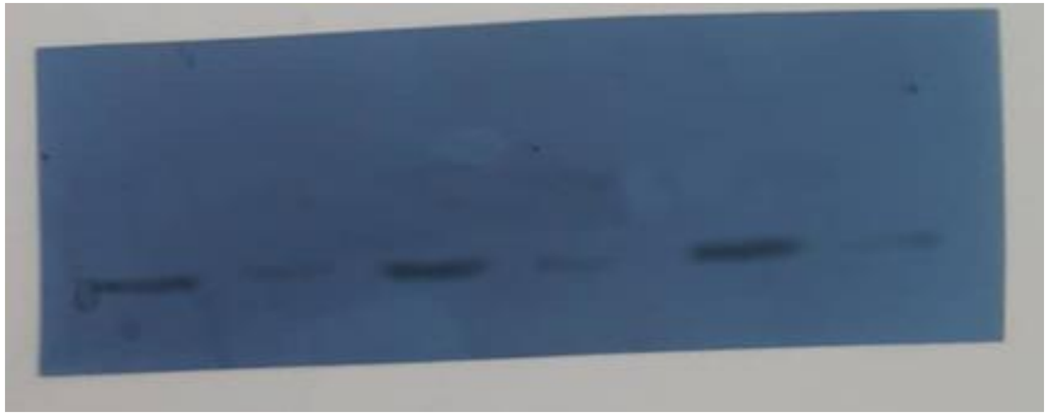

GAPDH

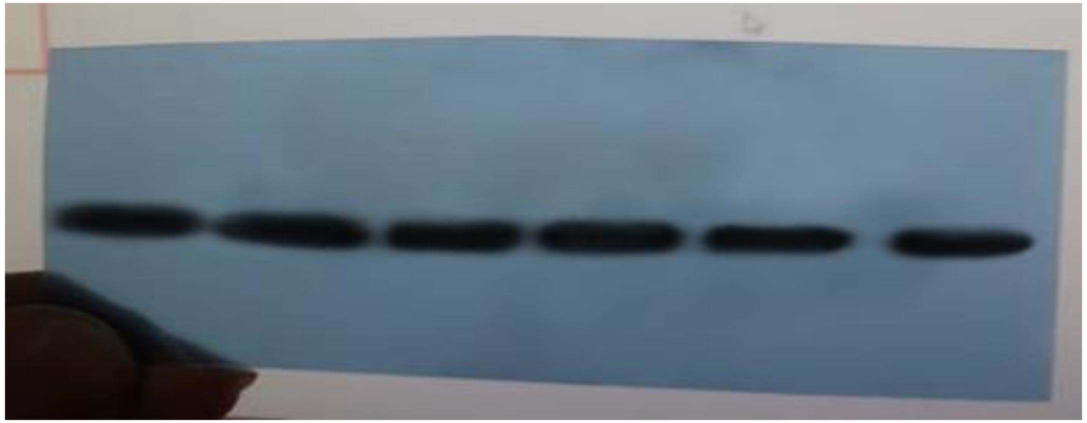

Supplement: Supplementary file 1 — Additional file 1. [file 12958_2021_758_MOESM1_ESM.pdf]
